# The Intersection of Cancer, Geriatrics, and Gerontology: A Multidisciplinary Approach Across the Cancer Continuum

**DOI:** 10.1093/geroni/igab046.1153

**Published:** 2021-12-17

**Authors:** Jessica Krok-Schoen

## Abstract

Despite the majority of cancer survivors being older adults, the connection between oncology, geriatrics, and gerontology remains unexplored. Our symposium will provide insights across the cancer continuum from prevention through survivorship as well as a comprehensive view of the connection between gerontological and geriatric factors in oncology. Specifically, we will discuss the biopsychosocial and behavioral factors among older adults with cancer, their effect on health outcomes, and how researchers and clinicians can intervene to improve health outcomes. The first abstract by Dr. Cadet found that despite a lack of knowledge of options and harms of cancer screening among older adults with low health literacy, there was a desire to understand more to better their health. The second abstract by Dr. Bhattacharyya found that older patients with cancer experience high levels of social isolation, loneliness, and fear that are heightened by individual and technology-based barriers to telehealth. The third abstract by Dr. Carroll found that breast cancer survivors with good sleep quality had less accelerated biological aging than those with sleep problems. The fourth abstract by Dr. Krok-Schoen utilized one of the largest datasets of older women, the Women’s Health Initiative, and found multiple gerontological and geriatric factors associated with physical activity among older female cancer survivors. Closing this symposium is Dr. Guida, a Program Director at the National Cancer Institute (NCI), who will profile the current research efforts, programmatic priorities, and current funding in aging and cancer. These multidisciplinary researchers and clinicians will provide a comprehensive symposium regarding geriatric oncology.

